# Comparison of antiangiogenic agents (ranibizumab, aflibercept, bevacizumab and ziv-aflibercept) in the therapeutic response to the exudative form of age-related macular degeneration according to the treat-and-extend protocol- true head-to-head study

**DOI:** 10.1186/s40942-024-00537-5

**Published:** 2024-02-02

**Authors:** Tereza Kanadani, Neiffer Rabelo, Denise Takahashi, Lucas Magalhães, Michel Farah

**Affiliations:** 1 Retina Institute, Belo Horizonte, Brazil; 2 Federal University of Sao Paulo, Sao Paulo, Brazil; 3 Nova Lima, Brazil

**Keywords:** Age-Related Macular Degeneration, Angiogenesis Inhibitors, Bevacizumab, Biomarkers, Clinical Protocols, Drug Therapy, Macular degeneration, Ranibizumab, Tomography Optical Coherence, Therapeutic Uses

## Abstract

**Purpose:**

To evaluate the structural and functional changes in eyes with neovascular age related macular degeneration (nAMD) in a real-world setting, using Treat and Extend protocol (T&E), comparing four antiangiogenic agents.

**Methods:**

Prospective, observational, case series study performed in 131 patients with the exudative form of nAMD. Patients were randomly assigned into four groups according to the antiangiogenic agent. During the first year, all eyes received at least 3 monthly intravitreal injections of antiangiogenic agents, and afterwards, were submitted to the T&E.

**Results:**

There was statistically significant difference (p < 0.05) between pre- and post-treatment in the best corrected visual acuity measurements by drug used. Patients who used aflibercept had significantly fewer injections than patients using the other drugs (mean = 9.03). No significant difference was observed between the drugs bevacizumab, ranibizumab and ziv-aflibercept. With regard to biomarkers, patients who used aflibercept and had lower baseline central retinal thickness, absence of hyperreflective foci and no subretinal hyperreflective material had the lowest number of injections.

**Conclusion:**

Results indicate that over 2 years, Intravitreal aflibercept on T&E provided better visual and anatomical improvements when compared to other drugs used in this study with significantly fewer injections.

## Introduction

Intravitreal therapy with vascular endothelial growth factor-inhibiting agents (anti-VEGF) is considered the gold standard treatment for exudative AMD [1]. However, some patients are non-responsive, showing resistance to treatment with anti-VEGF agents [2].

Although fixed treatment regimens achieved good visual results in clinical trials with monthly injections, in daily practice, the multiple office visits represent an overwhelming management challenge for patients and their families, and an increase in costs, which may be a barrier for the long-term patient compliance [3, 4]. This may be why other studies have shown that eyes with neovascular AMD (nAMD) are often treated with less anti-VEGF injections that necessary [5].

In order to address this issue, flexible treatment protocols are more often being adopted in daily practice, such as pro re nata (PRN or "Treat if necessary") and treat an extend (T&E). Regarding PRN protocol, despite the association with minor number of intravitreal injections, there was no reduction on the number of visits, considering the monthly examinations required. In the search for the ideal protocol—one that reduces both visits and injections, T&E may be an optimized alternative. This flexible treatment method is already known to be associated with better long-term visual outcomes, better disease stability and better patient compliance. For that, is the election strategy in several guidelines of multiple clinical trials. Additionally, many surveys have shown that T&E is the main regimen in daily practice [6–11].

Still, it isn’t clear if there are any differences between the use of T&E protocol with available anti-VEGF agents (ranibizumab, aflibercept, bevacizumab and ziv-aflibercept). Ziv-aflibercept (Zaltrap; Sanofi-Aventis US LLC, Bridgewater, NJ, and Regeneron Pharmaceuticals) is a systemic chemotherapy approved by the Food and Drug Administration (FDA) for the treatment of metastatic colorectal cancer. Ziv-aflibercept contains a fusion protein called aflibercept, the same one that the FDA has approved for intraocular use but with a higher osmolarity [12]. Because of the lower cost of intravitreal ziv-aflibercept compared with aflibercept and no proven toxicity, the former one gained global attention for its potential intraocular use to treat various retinal diseases, especially in developing countries [13].

The aim of this study was to assess the structural and functional changes in eyes with nAMD in a real-world setting, using T&E protocol, comparing four antiangiogenic agents. It is worth noting that this is the first study where such a comparison between antiangiogenic agents was performed.

## Material and methods

A prospective, observational, case series study was performed in 131 patients with the exudative form of AMD who were seen at the Retina and Vitreous Department of the Retina Institute, Belo Horizonte, MG. The patients were randomly assigned into four groups according to the antiangiogenic agent from September 2020 to August 2022, during the COVID-19 pandemic period. The study protocol was approved by the Ethics Committee of Department of Ophthalmology, Federal University of São Paulo, Brazil and was fullfilled in respect to the Declaration of Helsinki proposition.

Anamnesis data were collected regarding age, sex and history of hypertension, hypercholesterolemia, smoking using a specific questionnaire. Ophthalmological examination data included best corrected visual acuity (BCVA) using the ETDRS table (Early Treatment of Diabetic Retinopathy Study), biomicroscopy of the anterior and posterior segments and indirect binocular ophthalmoscopy. The imaging examinations performed were: color and red-free fundus photographs and fluorescein angiography (FA), which were obtained using the TRC-50IX Retinal Camera/IMAGEnet 2000 instrument (Topcon, Tokyo, Japan), and spectral domain optical coherence tomography (OCT), whose images were captured using the 3D OCT-2000 (Topcon).

Clinical ophthalmological examination, color fundus photographs, FA and OCT were performed in all patients in the study. Indocyanine green angiography was performed in patients showing signs considered suspicious for type 1 aneurysmal neovascularization or polypoidal choroidal vasculopathy (PCV), which included the presence of exudative maculopathy, associated with one of the following findings:—subretinal nodule, red–orange, detected on clinical ophthalmic examination;—subretinal hemorrhage;—retinal pigment epithelium (RPE) detachment displaying a notch or notch identified on AF; or—RPE with an ogive appearance, identified on OCT.

The BVCA was tested by a blinded operator. On the other hand, the Ophtalmological examination (biomicroscopy and indirect binocular ophthalmoscopy) and the imaging examinations and measurements were executed by a blinded retinal specialist (R.M.A).

Data were analyzed before and after antiangiogenic therapy, including age, race, sex, affected eye, duration of symptoms, smoking, hypercholesterolemia, hypertension, BCVA, IOP (intraocular pressure) and spectral domain tomographic measurements (central retinal thickness—CRT, intraretinal fluid—IRF, subretinal fluid—SRF, hyperreflective foci—HRF, subretinal hyperreflective material—SHRM, retinal pigment epithelium detachment—PED, and subfoveal thickness of the choroid—SChT). PED was classified as serous if optically empty, fibrovascular if non-homogeneous material was present and with a density greater than that of fluid between the retinal pigment epithelium (RPE) and Bruch's membrane, or mixed if there were characteristics of both.

### Anti-vascular endothelial growth factor (anti-VEGF) treatment

Intravitreal injections of antiangiogenic agents were with 1.25 mg/0.1 ml of bevacizumab, 0.5 mg/0.05 ml of Ranibizumab, Aflibercept 2.0 mg/0.05 ml and Ziv-aflibercept (0.07 ml or 0.08 ml of 100 mg/4 ml). During the first year, all eyes received at least 3 monthly intravitreal injections of antiangiogenic agents, and afterwards, they were submitted to the T&E protocol. It is important to enlighten that the patients were submitted to the injections with no charge.

### Treat-and-extend protocol—dose interval criteria

The dose interval was guided by the T&E protocol; interval of intravitreal injections was progressively elongated by two weeks until a maximum of twelve weeks if the was no suggestion of neovascular activity. On the contrary, if observed any sign of minor recurrence the interval was decreased by two weeks. Moreover, if a major recurrence was detected, the interval was further reduced by two weeks—returning to monthly treatment. Minor and major recurrences characterization were adapted from the literature. The first one indicated the presence of discrete intraretinal fluid without vision loss or foveal hemorrhage, while the second was associated with existence of intraretinal or subretinal fluid with vision loss of > 6 letters and/or foveal hemorrhage [6–11].

### Best corrected visual acuity and tomographic measurements

BCVA and the tomographic measurements CRT, IRF, SRF, SHRM, HRF and PED were measured before and 3, 6, 9, 12 and 24 months after the intravitreal injections.

### Inclusion criteria

(a) Age over 55 years; (b) diagnosis of wet AMD, including all subtypes; (c) indication of treatment with bevacizumab, ranibizumab, aflibercept or ziv-aflibercept in the affected eye; (d) previous antiangiogenic treatment up to 4 months before inclusion in the study; and (e) minimum follow-up of 2 years.

### Exclusion criteria

(a) Choroidal neovascularization secondary to other causes; (b) concomitant inflammatory eye diseases; (c) eyes submitted to vitrectomy; d) eyes with other conditions that could affect the vitreomacular interface, such as vascular retinal diseases, pathological myopia and diabetic retinopathy; and (e) presence of macular scar.

### Wet AMD subtypes

Eyes with wet AMD were classified as having "typical" nAMD (macular neovascular membranes type 1 and 2), aneurysmal type 1 neovascularization (PCV) or type 3 macular neovascularization (retinal angiomatous proliferation—RAP).

Patient data were evaluated by two retinal specialists. In case of divergence in the classification made by the two specialists, they met, discussed the data and reached a consensus.

Patients with unilateral active nAMD had the contralateral eye evaluated and classified according to the 5-level scale proposed by the clinical staging system for age-related macular degeneration in AREDS study [14].

### Statistical analysis

Categorical data were assessed using the χ2 or Fisher test. The study data were tested for a normal distribution using the Shapiro- Wilk test, which revealed that data were not normally distributed.

As a result, we used a nonparametric test for comparison of continuous variables between groups (Mann–Whitney test). In addition, the Wilcoxon test was used to compare follow-up and base- line data within a treatment group.

The data with normal distribution were presented as mean ± standard deviation (SD), while the data without normal distribution were given as the median (interquartile range [IQR]). Qualitative variables were assessed by the Pearson chi-square test or Fisher’s exact test. A p value of less than 0.05 was considered statistically significant.

## Results

One hundred and thirty-one eyes with nAMD with BCVA between 78 and 18 (Snellen equivalent, 20/32 and 20/400) from 120 patients were randomly assigned to the loading dose with aflibercept, ranibizumab, ziv-aflibercept and bevacizumab; afterwards, the patients were submitted to the T&E protocol. It is important to describe that 6, 6, 4 and 4 patients of the respective groups: bevacizumab, ranibizumab, ziv-aflibercept and aflibercept were excluded because of loss of follow up (10 of these 20 patients died of COVID during de pandemic). Baseline demographics were well balanced between the groups (Table 1).

**Table 1:** Baseline characteristics

Variables	Drug used							
	*Aflibercept*		*Bevacizumab*		*Ranibizumab*		*Ziv-aflibercept*	
	n	%	n	%	n	%	n	%
Age	(n = 33)		(n = 32)		(n = 33)		(n = 33)	
Mean ± SD	77,7 ± 8,4		80,0 ± 6,9		77,3 ± 6,4		74,5 ± 7,8	
Median (Q_1_–Q_3_)	78,0 (70,0–85,5)		80,5 (75,3–86,0)		78,0 (73,5–82,0)		76,0 (67,5–80,5)	
Mín–Máx	60,0–90,0		63,0–90,0		63,0–88,0		59,0–87,0	
Sex								
Male	13	39,4	7	21,9	10	30,3	15	45,5
Female	20	60,6	25	78,1	23	69,7	18	54,5
TOTAL	**33**	**100,0**	**32**	**100,0**	**33**	**100,0**	**33**	**100,0**
Race								
White	29	87,9	24	75,0	27	81,8	30	90,9
Brown	0	0,0	2	6,3	2	6,1	0	0,0
Black	4	12,1	6	18,7	4	12,1	3	9,1
TOTAL	**33**	**100,0**	**32**	**100,0**	**33**	**100,0**	**33**	**100,0**
Smoking								
Yes	8	24,2	10	31,3	12	36,4	12	32,1
No	25	75,8	22	68,7	21	63,6	21	67,9
TOTAL	**33**	**100,0**	**32**	**100,0**	**33**	**100,0**	**33**	**100,0**

Variables	Drug used							
	*Aflibercept*		*Bevacizumab*		*Ranibizumab*		*Ziv-aflibercept*	
	n	%	n	%	n	%	n	%
Hypercholesterolemia								
Yes	18	54,5	18	56,2	23	69,7	21	63,6
No	15	45,5	14	43,8	10	30,3	12	36,4
TOTAL	**33**	**100,0**	**32**	**100,0**	**33**	**100,0**	**33**	**100,0**
Hypertension								
Yes	31	93,9	28	87,5	30	90,9	31	93,9
No	2	6,1	4	12,5	3	9,1	2	6,1
TOTAL	**33**	**100,0**	**32**	**100,0**	**33**	**100,0**	**33**	**100,0**
Time of symptoms (months)	(n = 33)		(n = 32)		(n = 33)		(n = 33)	
Mean ± SD	15,7 ± 21,1		12,9 ± 12,4		23,2 ± 27,9		14,5 ± 17,3	
Median (Q_1_–Q_3_)	6,0 (4,0–12,0)		6,5 (4,3–21,0)		12,0 (4,0–24,0)		6,0 (3,0–18,0)	
Mín–Máx	1,0–96,0		3,0–48,0		3,0–108,0		2,0–60,0	
Previous treatment								
Yes	11	33,3	10	31,3	16	48,5	15	39,7
No	22	66,7	22	68,7	17	51,5	18	60,3
TOTAL	**33**	**100,0**	**32**	**100,0**	**33**	**100,0**	**33**	**100,0**

Variables	Drug used							
	*Aflibercept*		*Bevacizumab*		*Ranibizumab*		*Ziv-aflibercept*	
	n	%	n	%	n	%	n	%
Baseline BCVA								
0,30 (20/40)	1	3,0	0	0,0	0	0,0	0	0,0
0,48 (20/60)	0	0,0	0	0,0	1	3,0	1	3,0
0,54 (20/70)	7	21,2	7	21,9	2	6,1	5	15,2
0,60 (20/80)	2	6,1	1	3,1	0	0,0	1	3,0
0,70 (20/100)	9	27,3	7	21,9	9	27,3	4	12,1
1,00 (20/200)	10	30,3	10	31,2	7	21,2	8	24,3
1,18 (20/300)	0	0,0	0	0,0	1	3,0	0	0,0
1,30 (20/400)	4	12,1	7	21,9	13	39,4	14	42,4
TOTAL	**33**	**100,0**	**32**	**100,0**	**33**	**100,0**	**33**	**100,0**
Baseline IOP								
9	0	0,0	0	0,0	0	0,0	2	6,1
10	2	6,1	5	15,6	1	3,0	0	0,0
11	6	18,2	9	28,1	5	15,2	9	27,3
12	4	12,1	4	12,5	5	15,2	5	15,2
13	9	27,3	5	15,6	6	18,2	5	15,2
14	3	9,1	2	6,3	6	18,2	1	3,0
15	1	3,0	2	6,3	6	18,2	1	3,0
16	3	9,1	5	15,6	3	9,1	6	18,2
17	4	12,1	0	0,0	0	0,0	3	9,1
18	1	3,0	0	0,0	0	0,0	1	3,0
19	0	0,0	0	0,0	1	3,0	0	0,0
TOTAL	**33**	**100,0**	**32**	**100,0**	**33**	**100,0**	**33**	**100,0**

Variables	Drug used							
	*Aflibercept*		*Bevacizumab*		*Ranibizumab*		*Ziv-aflibercept*	
	n	%	n	%	n	%	n	%
Baseline IOP	(n = 33)		(n = 32)		(n = 33)		(n = 33)	
Mean ± SD	13,4 ± 2,3		12,5 ± 2,1		13,5 ± 1,9		13,2 ± 2,5	
Median (Q_1_–Q_3_)	13,0 (11,5–15,5)		12,0 (11,0–14,0)		13,0 (12,0–15,0)		13,0 (11,0–16,0)	
Mín–Máx	10,0–18,0		10,0–16,0		10,0–19,0		9,0–18,0	
Baseline CTR	(n = 33)		(n = 32)		(n = 33)		(n = 33)	
Mean ± SD	432 ± 73		470 ± 81		449 ± 73		469 ± 75	
Median (Q_1_–Q_3_)	413 (383–490)		477 (397–536)		450 (393–493)		464 (400–526)	
Mín–Máx	285–587		337–631		342–631		332–610	
Baseline IRF								
Yes	33	100,0	32	100,0	33	100,0	33	100,0
No	0	0,0	0	0,0	0	0,0	0	0,0
TOTAL	**33**	**100,0**	**32**	**100,0**	**33**	**100,0**	**33**	**100,0**
Baseline SRF								
Yes	33	100,0	30	93,7	31	93,9	33	100,0
No	0	0,0	2	6,3	2	6,1	0	0,0
TOTAL	**33**	**100,0**	**32**	**100,0**	**33**	**100,0**	**33**	**100,0**

Those values mean the total of each variable, so we would like to emphasize them. That's why they are bold.

### Best corrected visual acuity outcomes

There was a statistically significant difference (p < 0.05) between pre- and post-treatment in the distribution of BCVA measurements by drug used, where the distribution of BCVA measurements after treatment was significantly lower than before treatment (Table 2).

**Table 2:** Best corrected visual acuity between the groups and in general

Drug	Time	n			p
			Mean ± SD	Median (Q_1_–Q_3_)	
Aflibercept					
	*Baseline*	33	0.81 ± 0.27	0.70 (0.57–1.00)	** < 0.001**
	*Final*	33	0.17 ± 0.06	0.18 (0.10–0.18)	Z = 5.020; *r* = 0.87
Bevacizumab					
	Baseline	32	0.89 ± 0.28	1.00 (0.63–1.00)	** < 0.001**
	*Final*	32	0.41 ± 0.20	0.54 (0.18–0.60)	Z = 4.688; *r* = 0.82
Ranibizumab					
	Baseline	33	1.00 ± 0.29	1.00 (0.70–1.30)	** < 0.001**
	*Final*	33	0.43 ± 0.19	0.54 (0.24–0.60)	Z = 4.946; *r* = 0.86
Ziv-aflibercept					
	Baseiline	33	0.99 ± 0.31	1.00 (0.70–1.30)	** < 0.001**
	*Final*	33	0.41 ± 0.22	0.40 (0.18–0.60)	Z = 4.806; *r* = 0.84

Note: p → *Wilcoxon’s test; r* → effect size

### Treat-and-extend protocol and number of injections

With regard to the number of patient injections (Table 3), the results showed a statistically significant difference (p < 0.001) between the drugs used. Patients who used aflibercept had significantly fewer injections than patients using the other drugs (mean = 9.03). In addition, no significant difference was observed between the drugs bevacizumab (mean = 10.06), ranibizumab (mean = 10.52) and ziv-aflibercept (mean = 10.06).

**Table 3:** Descriptive and comparative analyses between the four drugs studied regarding the number of intravitreal injections after the loading dose

Drug	n		P
		Mean ± SD	
*Aflibercept* (1)	33	9.03 ± 0.17	** < 0.001** (*F*_3. 127_ = 12.267) 1 < (2 = 4 = 3)
*Bevacizumab* (2)	32	10.06 ± 1.13	
*Ranibizumab* (3)	33	10.52 ± 1.33	
*Ziv-aflibercept* (4)	33	10.06 ± 1.09	
General	131	9.92 ± 1.16	

Notes: *Welch*’s test

Table 4 shows a statistically significant difference (p < 0.001) between the drugs in terms of the percentage of injections, per patient, in periods of 90 days. Patients treated with aflibercept had a percentage of injections in periods of 90 days that was significantly higher compared to patients using the other drugs (bevacizumab, ranibizumab and ziv-aflibercept). In addition, no significant difference was observed in the percentage of injections in periods of 90 days between these three drugs.

**Table 4:** Descriptive and comparative analysis between the four drugs regarding the percentage of injections made, per patient, in periods of 90 days

Drug	n	(%)	
		Mean ± SD	Median (Q_1_–Q_3_)
Aflibercept	33	49.7 ± 1.4	50.0 (50.0–50.0)
Bevacizumab	32	28.1 ± 19.1	33.3 (8.3–50.0)
Ranibizumab	33	26.3 ± 21.2	25.0 (0.0–50.0)
Ziv-aflibercept	33	30.6 ± 19.2	33.3 (16.7–50.0)
		**p < 0.001** (H_3_ = 34.594; Z = 4.335 and *r* = 0.75) Aflib > (Beva = Rani = Ziv)	

Note: p → *Kruskal–Wallis* test

### OCT biomarkers and treatment outcomes

Anatomic improvements achieved through month 12 were maintained through month 24. At month 24, there was no significant difference in mean CRT between the groups. Regarding OCT biomarkers, patients who used aflibercept and had lower baseline CRT, absence of HRF and no SHRM had the lowest number of injections (Table 5).

**Table 5:** Multivariate linear regression analysis with the variables related to the number of injections

Variables	Non-standardized coefficient	Standardized coefficient	Assessment parameters		
	B	β	T	p	VIF
Drug					
Aflibercept	0	–	–	–	–
Bevacizumab	0.852	0.318	3.600	** < 0.001**	1.6
Ranibizumab	1.309	0.493	5.608	** < 0.001**	1.6
Ziv-aflibercept	0.853	0.321	3.676	** < 0.001**	1.6
Baseline CRT	0.002	0.131	1.718	*0.088*	1.2
HRF					
No	0	–	–	–	–
Yes	0.781	0.253	3.515	**0.001**	1.1
SHRM					
No	0	–	–	–	–
Yes	0.520	0.212	2.734	**0.007**	1.2

Notes: R^2^ = 38.9%, R^2^_adjusted_ = 35.9%, R = 0.623, Durbin-Watson = 2.12

### Adverse events

Two patients in the cohort had unscheduled visits in the first 2 years of the study because of adverse events, and no patients had more than 1 unscheduled visit. Serious ocular adverse events in year 2 of the TRUE HEAD-TO-HEAD STUDY included progressive macular atrophy and a subretinal hemorrhage. There were no cases of endophthalmitis or intraocular inflammation.

## Discussion

The T&E dosing regimen is the most commonly used treatment method for nAMD worldwide and is used by more than 77% of north American retinal at the time, as determined by the American Society of Retina Specialists 2014 PAT Survey [15]. Real-life studies aim at establishing a treatment protocol that optimizes both the number of treatment visits and injections, to decrease the financial burden on the healthcare system and improve patient compliance while maintaining visual outcomes. To our knowledge, this is the first study of a T&E dosing regimen comparing four types of anti-VEGF drugs in a setting of nAMD in real-world practice during the COVID-19 pandemic.

The fourth and newest drug present in this study is the ziv-aflibercept. This drug accommodates the same aflibercept (VEGF-trap) molecule, but with a higher osmolarity (1000 mOsm/kg vs 300 mOsm/kg) [16]. Initially, there was a preoccupation about cytotoxicity and long-term safety of intravitreal ziv-aflibercept, that have not been proved to be correct, after multiple publications have not identified adverse ocular and systemic side effects [16]. Information of various authors suggest that ziv-aflibercept may be as cost effective as bevacizumab, turning it into a both alternative and attractive treatment option in low- and middle-income countries, like Brazil [16].

A study by Rodrigues and colleagues attempted to compare real-life results of aflibercept and ranibizumab in patients with nAMD. This was a retrospective review of patients with nAMD who were treatment-naïve and receiving a fixed dosing regimen of either aflibercept or ranibizumab [17]. At the 12-month follow-up, there was no statistically significant difference in the change in BCVA between the two groups (*p* = 0.121), but the change in CRT was significantly better in the aflibercept group (− 142.2 versus − 51.5, p = 0.011), showing that while visual results were comparable between the two groups, the anatomical results were better with aflibercept [17]. Compared with this current study that was a direct prospective comparison between four anti-VEGF agents in a T&E dosing regimen, there was a statistically significant difference (p < 0.05) between pre- and post-treatment in the distribution of BCVA measurements by drug used, where the distribution of BCVA measurements after treatment was significantly lower than pre-treatment and the change in BCVA was better in the aflibercept group. At month 24, the changes in CRT did not differ significantly between the four treatment groups (p > 0.05). These results display that aflibercept maintains good visual outcomes in nAMD with a T&E protocol after two years of follow-up in a real-life setting.

In both the current and LUCAS studies [18], treatment intervals were lengthened progressively by 2 weeks until recurrent exudative disease was identified, at which point the interval was shortened by 2-week increments until a dry macula was reestablished. In a TRUE HEAD-TO-HEAD STUDY, the current study, patients treated with aflibercept had a percentage of injections during periods of twelve weeks significantly higher than the patients using the other drugs (bevacizumab, ranibizumab and ziv-aflibercept). In addition, no significant difference was observed in the percentage of injections during periods of twelve weeks between these three drugs.

Despite excellent outcomes in the current trial, some patients had substantial vision loss. One patient lost 20 ETDRS letters because of progressive macular atrophy. Although anti-VEGF agents are highly effective in nAMD, their role in the development or progression of macular atrophy is known [19]. This particular case was remarkable because macular atrophy progressed rapidly and the interval between anti-VEGF treatments concurrently was maximally extended.

BVCA preservation or improvement in nAMD patients was shown be associated with important prognostic factors, such as the quantity of injections, clinical appointments and OCT biomarkers. [20] This study had been designed to assess treatment differences regarding the change in BCVA and CRT between the four groups from baseline to 24 months as the primary outcome measures. The number of injections at 24 months was defined as a secondary objective. All patients received three initial monthly doses followed by an extension phase, during which the interval between subsequent injections was adjusted by 2 weeks within a range of a minimum of 4 weeks and a maximum of 12 weeks between administrations. About the number of anti-VEGF injections after the loading dose, there was a statistically significant difference between the drugs used (p < 0.001). Patients in the aflibercept group had significantly fewer injections than the patients using the other drugs (mean = 9.03). Additionally, no significant difference was observed between bevacizumab (mean = 10.06), ranibizumab (mean = 10.52) and ziv-aflibercept (mean = 10.06). Regarding OCT biomarkers, patients who used aflibercept, with lower baseline CRT, absence of HRF and no SHRM had the lowest quantity of injections. Furthermore, patients treated with IVT-AFL (intravitreal aflibercept) in the RIVAL study [21] received more intensive treatment than described in the ALTAIR trial [22] assessing IVT-AFL regimens (17 injections vs 10.4 injections at 104 and 96 weeks, respectively) and combined IVT- AFL Q8W arms from the VIEW 1 and 2 trials [23, 24] (17 injections vs 11.2 injections at 104 and 96 weeks, respectively), which may constitute overtreatment. While the RIVAL study is the only clinical trial comparing IVT-AFL and ranibizumab in an identical proactive treatment regimen, described as T&E, the current study is unique because there was a comparison between four different anti-VEGF agents in a real-world practice.

The strengths of a TRUE HEAD-TO-HEAD study are its prospective design involving well-defined patient cohorts and high protocol compliance even during the pandemic period, in which despite the restriction time, we were able to analyze the outcome of the treatment used without causing any harm to the patients well being. This study is the first to compare 4 different drugs, 2 of which off-label for nAMD, in the T&E protocol in a real-world scenario. The limitations of this study include the inherent variability of follow-up intervals using a T&E protocol, as well as the limited number of patients.

The results of this comparison indicate that over 2 years, IVT-AFL T&E provided better visual and anatomical improvements when compared to other drugs used in this study with a lower treatment burden based on significantly fewer injections.

## Data Availability

The data supporting the results of this study will be made available by the corresponding author, T.C.M.K, upon request.
